# Getting to the heart of an unusual kinetochore

**DOI:** 10.1098/rsob.160040

**Published:** 2016-04-13

**Authors:** Martin R. Singleton

**Affiliations:** Structural Biology of Chromosome Segregation Laboratory, The Francis Crick Institute, 44 Lincoln's Inn Fields, London WC2A 3LY, UK

## Abstract

The Mis12 complex forms the central scaffold of the kinetochore and serves to bridge the chromatin and microtubule-binding activities of the inner and outer layers, respectively. Two recent studies provide new structural insights into the formation of this complex, and highlight some intriguing adaptations found in the *Drosophila* kinetochore.

Chromosome segregation is a fascinating and highly complex process. The logistical demands of translocating the huge, condensed polymers that constitute sister chromatids to opposite poles of the cell alone is a challenge. To ensure that this occurs in an accurate and timely manner adds an additional layer of complexity [1]. The kinetochore is one of the key cellular structures that impacts directly on both processes. The direct *in situ* visualizations of these structures by electron microscopy (e.g. [2]) have revealed some general principles of their organization, and led to the formulation of a popular model that depicts the kinetochore as a flattened disc, with an inner, chromatin-proximal layer connected to an outer microtubule-binding interface. Defining the proteins that form these layers and determining their individual structures and the relationships between them is an ongoing task, and many recent studies have begun to allow us to build a more precise picture of how the kinetochore is formed (reviewed in [3–5]).

Kinetochores are built upon centromeric chromatin, which is distinguished by the presence of nucleosomes containing the histone H3 variant, CENP-A [6]. Both the CENP-A and canonical nucleosomes interact with the proteins of the inner kinetochore, collectively known as the CCAN (constitutive centromere-associated network) [7,8]. This in turn links to the KMN (Knl1 complex, Mis12 complex, Ndc80 complex) network responsible for binding microtubules and recruiting checkpoint proteins to the kinetochore [9]. Functional dissections of the network have shown that the Ndc80 complex (Ndc80C, consisting of the proteins Ndc80, Nuf2, Spc24 and Spc25) forms the principal microtubule-binding site via calponin homology domains in Nuf2 and Ndc80 [10,11]. Knl1, which forms a dimer with Zwint, provides a platform for recruitment of a variety of checkpoint components [12–14]. Finally, the Mis12 complex (Mis12C, consisting of Mis12, Dsn1, Nsl1, Nnf1) provides the central scaffold to coordinate these activities and link them to the CCAN [9,15,16] (figure 1*a*).

**Figure 1.**
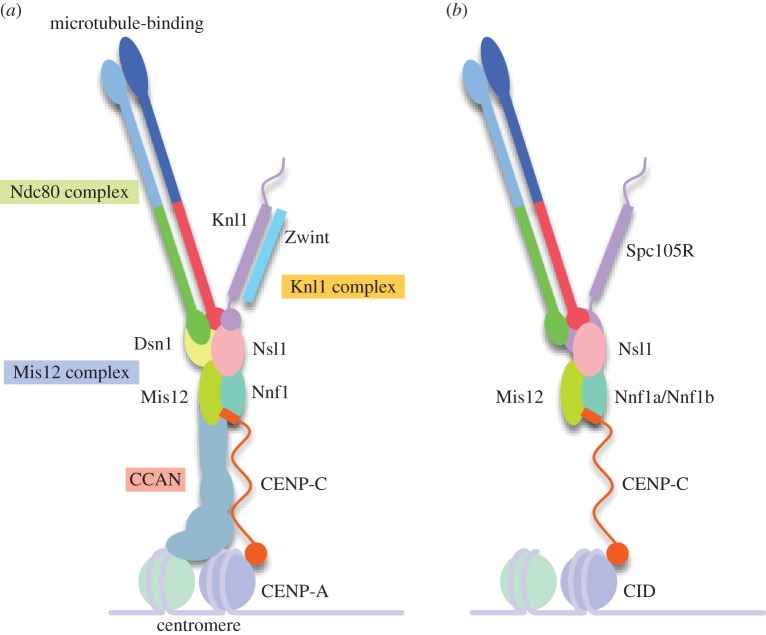
Schematic diagrams of the kinetochore, showing the path from the centromere to the microtubule-binding site at the tip of the Ndc80 complex. This basic structure is presumed to be a repeated unit in cells. (*a*) The main components in the human kinetochore. (*b*) The organization in *Drosophila*, highlighting the lack of the CCAN, Zwint subunit of Knl1/Spc105R and Dsn1.

This general organization appears to be widely conserved. However, some organisms (such as *Drosophila*) appear to almost entirely dispense with the CCAN [17], just maintaining CENP-C as the sole connection to chromatin. Uniquely, in *Drosophila*, the Mis12 complex has also lost the Dsn1 subunit. Instead, the C-terminal of the Knl1 homologue, Spc105R, seems to have evolved to substitute for Dsn1, and is required for both full assembly of the Mis12 complex and its recruitment to the kinetochore [18,19] (figure 1*b*). Furthermore, the Nnf1 protein exists as a pair of paralogues, Nnf1a and Nnf1b, in contrast to other species [20,21]. Why these differences exist, and their implications for the structure and function of the complex, are unclear.

Structural studies of the kinetochore have proved challenging. The compositional peculiarities of many individual kinetochore proteins, which often contain long stretches of coiled-coil or intrinsically disordered sequence, make them difficult to crystallize for X-ray analysis. Furthermore, the larger complexes are often highly elongated and/or flexible, which limits the power of electron microscopy studies. The Mis12 complex is a case in point. Although the overall architecture has been defined [22–24], high-resolution structural information is still lacking and the detailed interactions between the constituent proteins are not fully understood.

Two papers in this issue of *Open Biology* address both these issues [25,26]. The authors present *in vitro* reconstitutions of the *Drosophila* Mis12 complex together with biophysical analyses to provide insights into the unique architecture of the *Drosophila* complex and more general details of the Mis12 complex as a whole.

Previous studies on the yeast and human Mis12 complex have shown that it may be subdivided into two dimers: one of Mis12–Nnf1 and one of Dsn1–Nsl1 [22–24]. Using reconstitution experiments, both groups show that the *Drosophila* complex retains a similar organization, with Mis12 and Nnf1 forming a tight, probably constitutive dimer (MN). Liu *et al*. [26] further show that this dimer may form with either Nnf1a or Nnf1b, leading to two distinct Mis12 complexes. Both groups also demonstrate that this core MN dimer can bind Nsl1 (referred to as Kmn1 in [26]), forming a stable trimer (MNN). Interestingly, negative stain electron microscopy pictures of this complex show that it forms an extended polarized structure, remarkably similar to its yeast and human counterparts, despite the absence of the Dsn1 subunit. Given that Dsn1 is the largest component in these complexes, and the other proteins are all of roughly similar size, the implication is that some or all of the proteins in the *Drosophila* complex are sufficiently extended to run the full length of the complex, and that this organization is probably a general feature. Analytical ultracentrifugation and size-exclusion chromatography results presented in both papers support this, and show that both the MN and MNN complexes are indeed highly elongated.

To map the finer details of the interactions within the complexes, complementary mass spectrometry (MS)-based techniques were used. The hydrogen/deuterium exchange (HDX-MS) approach taken by Richter *et al*. [25] relies on the fact that the amide protons of the peptide backbone typically have a retarded rate of exchange with the solvent when that section of the protein is involved in contacts with another molecule. Regions of the protein protected from exchange (as determined by the hydrogen/deuterium mass ratio) are therefore indicative of an interaction interface. Another approach, taken by Liu *et al*. [26], is to cross-link the proteins using an amine-directed linker which will connect lysine side-chains within a given distance (XL-MS). Protease digestion of the cross-linked complex, followed by MS identification of the cross-linked peptides, allows a map of interacting regions to be constructed.

Using these techniques, both groups found that the C-termini of Mis12 and Nnf1 interacted strongly, a finding that was subsequently confirmed by deletion analyses. The interacting region lies within a predicted coiled-coil domain. By modelling the Mis12 and Nnf1 sequences onto a known coiled-coil template, Richter *et al*. were able to synthesize peptides corresponding to the interacting regions that maximized the number of leucine–leucine interactions, known to be important for coiled-coil dimerization. Using these peptides, the interacting regions were further narrowed down by nuclear magnetic resonance studies. Finally, the presumptive critical leucine residues were mutated and the resulting constructs expressed in cell lines. Pull-down experiments followed by mass spectrometry (AP-MS) established the importance of the residues for the interaction and support the notion that this C-terminal coiled-coil is the main dimerization element.

How then does the MN complex interact with other components of the Mis12 complex and the rest of the kinetochore? Following the approaches used to examine the dimerization of Mis12–Nnf1, both groups analysed the interaction of the MN dimer with the third member of the complex, Nsl1/Kmn1, and the CCAN component, CENP-C. Both HDX-MS and XL-MS experiments revealed evidence of extensive interactions between Mis12/Nnf1 and the central region of Nsl1, which was subsequently demonstrated to be sufficient to mediate the interaction in pull-downs [25]. While HDX-MS was indicative of Nsl1 binding to the C-terminal of the MN dimer, the XL-MS analysis also revealed the existence of additional cross-links throughout the length of the proteins. This may result from the fact that the cross-linking agent can capture sites that are separated by a larger distance than is compatible with protection of proton exchange in the HDX-MS assays, and may reveal more transient or weaker binding sites.

The interaction between the Mis12 complex and CENP-C has previously been described in *Drosophila* and humans, and involves a relatively short section of the N-terminal of CENP-C [15,16]. Interestingly, CENP-C itself is highly divergent in both length and sequence, and a clear consensus Mis12 interaction motif has not been identified. Using deletion experiments, both studies identify a short N-terminal stretch of about 100 residues sufficient to robustly mediate the interaction with the Mis12 complex. Richter *et al*. [25] further pinpoint two conserved phenylalanine residues that appear essential for binding. In the partner MN complex, a set of conserved phenylalanine residues in the N-terminal of Mis12 and several hydrophobic residues in the N-terminal of Nnf1 were identified on the basis of experimental evidence and sequence conservation [25,26]. AP-MS or *in vitro* reconstitution experiments using appropriate mutant proteins conformed the essentiality of these hydrophobic interactions for complex formation.

The ‘missing' protein in the *Drosophila* Mis12 complex, Dsn1, appears to have been functionally replaced by the C-terminal of Spc105R [18]. In other species, this domain forms a so-called double-RWD (DRWD) fold [27] that is widely distributed among kinetochore proteins [28]. A crystal structure of the human Knl1 DRWD domain shows it binding directly to the C-terminal tail of Nsl1 [29]. Sequence analysis of the *Drosophila* Spc105R fails to show clear evidence of the existence of a similar domain, though it is possible it could exist in a highly diverged form. Nevertheless, it is shown that the extreme C-terminal of *Drosophila* Spc105R is indeed able to bind the Mis12 complex [26], but the structural relationship to that determined in other species remains unclear.

The overall picture that emerges from these studies is that of an extended, modular structure, in which Mis12 and Nnf1 form a key platform with a centromere-binding (N-terminal) and outer kinetochore-binding (C-terminal) end. Dimerization of the MN complex occurs via a central coiled-coil interaction and is absolutely required for recruitment of other members of the complex. In a sense, the centromere-binding and outer kinetochore-binding activities are separable, in that mutations that impair interaction with CENP-C (and hence the centromere) do not affect the formation of the complex *per se* [25]. The extreme modular nature of the complex and probable flexibility may well be important to accommodate the highly variable geometry of the microtubule-binding sites at one end, and underlying chromatin at the other. It is also notable that the stripped-down nature of the *Drosophila* kinetochore implies that all the spindle forces are transmitted to the chromosome through a single Mis12C–CENP-C interaction. Whether this is indeed the case will require further study.

These two studies have provided new, atomic-level insights into the formation of this still mysterious complex. However, many questions remain. The existence of two paralogues of Nnf1 remains unexplained. Although studies have suggested differing expression patterns [20,21], reconstituted complexes containing each variant appear indistinguishable, at least *in vitro* [26]. Furthermore, the mode of binding of the two other members of the KMN network (Spc105R and Ndc80C) to Mis12C are still not clearly understood, nor whether Spc105R contributes to the integrity of the complex by structurally substituting for Dsn1. It is to be hoped that further studies will address these specific problems and shed new light onto the organization of the KMN network as a whole. Finally, the results reported here are a nice reminder that useful structural information can be obtained on systems even when higher-profile methods of structural analysis prove wanting.
